# Elevated neutrophyl-to-lymphocyte ratioand smoking are associated with chronic total occlusion in patients with ST elevation myocardial infarction

**DOI:** 10.1186/s12872-023-03680-3

**Published:** 2024-01-03

**Authors:** Fenandri Fadillah Fedrizal, Ika Prasetya Wijaya, Murdani Abdullah, Muhammad Yamin

**Affiliations:** 1 Division of Cardiology, Department of Internal Medicine, Faculty of Medicine, Dr. Cipto Mangunkusumo Hospital, Universitas Indonesia, Jakarta, Indonesia; 2 Department of Internal Medicine, Faculty of Medicine, Dr. Cipto Mangunkusumo Hospital, Universitas Indonesia, Jakarta, Indonesia; 3 Unit Epidemiology, Department of Internal Medicine, Faculty of Medicine, Dr. Cipto Mangunkusumo Hospital, Universitas Indonesia, Jakarta, Indonesia

**Keywords:** Coronary artery Disease, Myocardial Infarction, Chronic total occlusion, Coronary angiography, Neutrophil-to-lymphocyte ratio

## Abstract

**Background:**

Atherosclerosis is a progressive disease characterized by the build-up of lipids and connective tissue in the large arteries. Some patients experience chronic total occlusion (CTO). Inflammation plays a key role in the development and complications of atherosclerosis. Neutrophil-to-lymphocyte ratio (NLR) is a marker of systemic inflammation associated with the development of acute coronary syndrome (ACS). We aimed to assess the relationship between NLR and CTO in ACS patients with ST elevated myocardial infarction (STEMI) in Indonesia.

**Methods:**

This cross-sectional study was performed with secondary data obtained from patient medical records at Cipto Mangunkusumo National Central Hospital, Jakarta. Inclusion criteria were patients with ACS and STEMI who underwent coronary angiography in 2015–2018.

**Results:**

A total of 98 patients were enrolled in the analysis. Most subjects with CTO were male, elderly (> 60), smoking, had no history of diabetes mellitus (DM) or hypertension, no family history of coronary heart disease (CHD), but had a history of ACS and had never consumed statin or antiplatelet medications. Bivariate logistic regression analysis revealed that male gender (PR = 1.820; 95%CI 0.871–3.805; *p* = 0.025) and smoking (PR = 1.781; 95%CI 1.028–3.086; *p* = 0.004) were significantly correlated with CTO. Receiver operator characteristic (ROC) curve revealed that higher NLR (≥ 6.42) could predict a CTO diagnosis with positive predictive value (PPV) of 91%. Multivariate analysis revealed that NLR was correlated with an 11.2-fold increase in occurrence of CTO (95%CI 3.250-38.303; *p* < 0.001). Additionally, smoking was correlated with a 7-fold increase in CTO (95% CI 1.791–30.508; *p* = 0.006).

**Conclusion:**

NLR value of ≥ 6.42 is potentially useful as a marker of CTO in STEMI patients. In addition, smoking increases the risk of CTO in ACS/STEMI patients.

**Supplementary Information:**

The online version contains supplementary material available at 10.1186/s12872-023-03680-3.

## Introduction

According to the World Health Organization (WHO), cardiovascular disease was the number one global killer in 2019, with 17.9 million deaths [1]. Similar conditions exist in Indonesia, as heart disease is the second leading cause of death after stroke. According to the 2018 Indonesian Health Survey (*RISKESDAS*), 1.5% of the population has been diagnosed with coronary artery disease (CAD) [2]. CAD is characterized by atherosclerosis in the coronary arteries, which can range in appearance from stable angina to acute coronary syndrome (ACS), such as unstable angina, ST elevated myocardial infarction (STEMI), and non-ST elevated myocardial infarction (NSTEMI). Chronic total occlusion (CTO) is a lesion with a duration of more than 3 months in which vessels show complete obstruction of blood flow on angiography or minimal penetration of contrast through the lesion, without changing the opacity of the distal vessels (becoming cloudier). Studies conducted in the 1990s reported CTO prevalence reaching 33–52% on routine angiography [3, 4]. However, several more recent studies have shown a downward trend, with CTO in 20–30% of patients with CAD [5, 6].

Atherosclerosis is a progressive disease characterized by the build-up of lipids and connective tissue in the large arteries. Inflammation plays a key role in the development and complications of atherosclerosis, beginning with endothelial dysfunction [7]. Neutrophils play a quintessential role in atherogenesis, thus they are inflammatory markers. Their infiltration into atherosclerotic plaques causes plaque disruption and increased platelet-neutrophil adhesion [8]. Furthermore, neutrophils can make plaques more susceptible to rupture through the release of proteolytic enzymes and super oxide free radicals. Neutrophils also secrete inflammatory mediators and have been associated with an acute inflammatory response to tissue injury [9]. The neutrophil-to-lymphocyte ratio (NLR) is a marker of systemic inflammation associated with the development of ACS [7]. Changes in the NLR occur when the balance between innate (neutrophils) and adaptive (lymphocytes) immunity is altered. STEMI patients exhibit increased neutrophil production in response to severe myocardial damage. Research has shown that the NLR increases over time in CAD patients and reaches a maximum around the time of ACS [10]. NLR is an affordable, simple, fast responding and easily accessible parameter with a high sensitivity [7]. Many studies have been conducted to evaluate the relationship between NLR and the incidence of CAD and ACS, but, to the best of our knowledge, no study has investigated the relationship between NLR and CTO in ACS patients, even more so in the Asian population. We aimed to determine the relationship between NLR and CTO in ACS patients with STEMI in Indonesia. In addition, we assessed for relationships between incidence of CTO in ACS patients and other cardiovascular risk factors such as age, gender, DM, hypertension, hyperlipidemia, history of antiplatelet and statin use, family history of CHD, and smoking.

## Methods

This cross-sectional study included all adult patients (18 years and above) who were diagnosed with ACS and STEMI and underwent coronary angiography in 2015–2018 at Cipto Mangunkusumo Hospital. Those who had a history of coronary bypass surgery, hematological disease, malignancy, severe liver and/or kidney disease, chronic or acute infections, autoimmune diseases, or incomplete medical records were excluded from the study.

We used a consecutive sampling technique to include subjects until reaching the minimum required sample size. Sample size was calculated using the formula for hypothesis tests for a two-sided population proportion. After specifying α = 0.05 and β = 0.02 (for 80% power), we calculated a total required sample size of 98 subjects. Data collected from medical records included NLR, age, gender, history of DM, hypertension, hyperlipidemia, previous ACS, family history of CAD, smoking status, and history of medication consumption (statin and antiplatelet). CTO lesion was defined as 100% occlusion of the coronary artery based on CT-angio or angiography with a minimum duration of 3 months. To minimise inter-individual variability, all subjects included in this study have similar clinical diagnosis i.e. chronic clinical syndrome and the majority of patients have comparable coronary risk factors for coronary artery diseases such as diabetes, hypertension, dyslipidemia, smoking, and obesity. Lesion included in this study (CTO lesion) is a chronic lesion which is considered stable and similar in its clinical course.

We performed statistical analyses with Stata version 14 software. Descriptive analysis was done to explore the characteristics of subjects. Numerical or continuous variables were reported as median (interquartile range/IQR), based on Kolmogorov-Smirnov test results. Categorical variables are presented as frequencies and percentages. The NLR cut-off point was analyzed by ROC curve for NLR in patients with CTO, area under the curve (AUC) value, and Hosmer-Lemeshow test. Model calibration was performed to ensure robustness. Bootstrapping techniques were used for internal validation. We repeated the entire modelling process, including variable selection in 1000 samples drawn with replacement from the original sample. Furthermore, the sensitivity, specificity, negative predictive value (NPV), positive predictive value (PPV), and likelihood ratio (LR) were calculated following the ROC curves for NLR. Statistical significance was set at *p* < 0.05. All variables were divided into two groups, with and without CTO. Bivariate analysis for NLR, age, gender, DM, hypertension, hyperlipidemia, history of antiplatelet and statin use, family history of CAD, and smoking was done with Chi-square test to assess for associations with chronic total occlusion. We included parameters with *p*-values > 0.25 in bivariate analysis into the multivariate analysis with backward logistic regression. Fit model Prevalence Ratio with 95% confidence intervals (CI) were calculated. A *p*-value < 0.05 was considered to be statistically significant for all calculations.

## Results

**Fig. 1:**
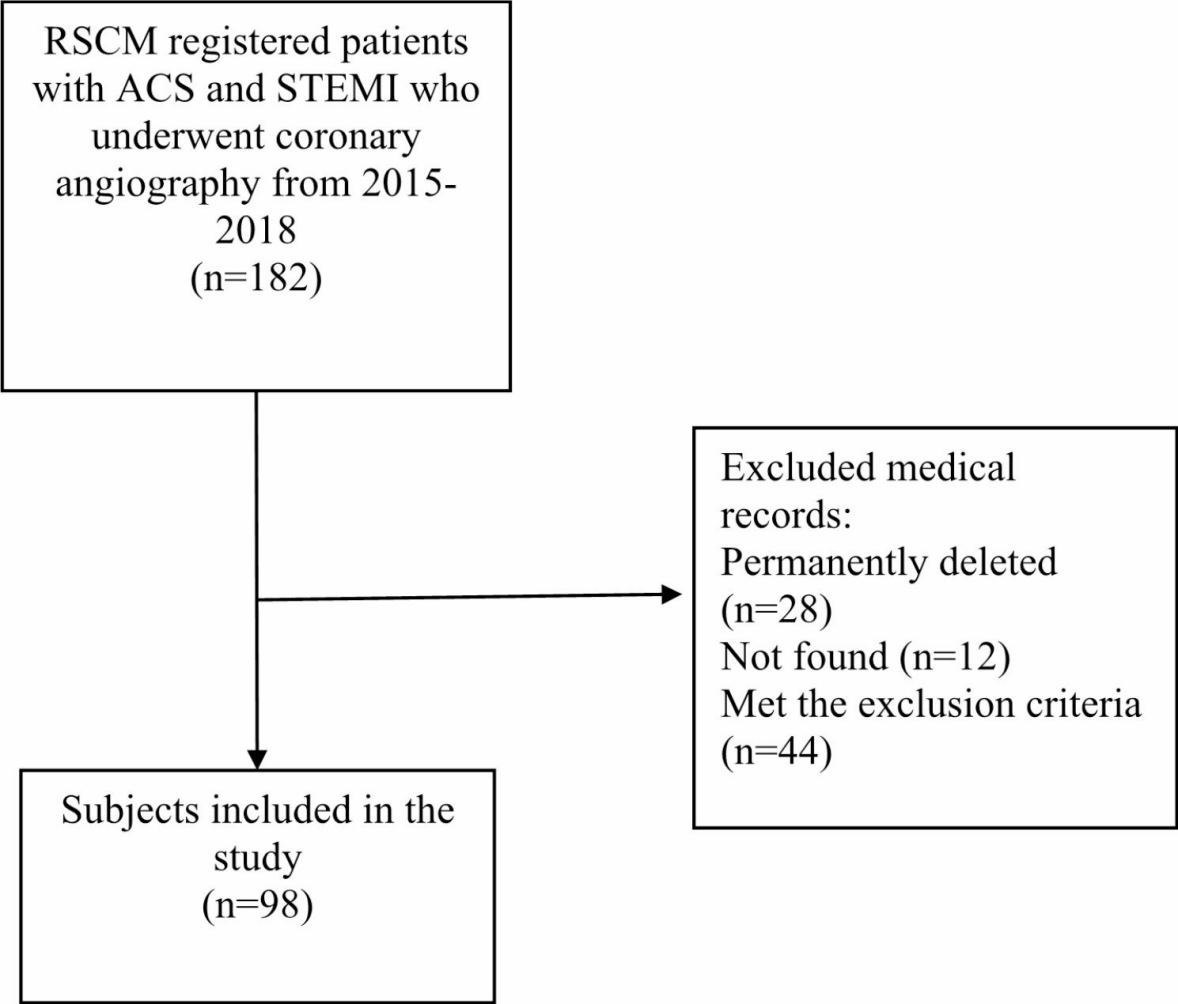
Study flow chart of subject inclusion

A total of 182 patients were registered as STEMI patients who underwent coronary angiography from 2015 to 2018 in Cipto Mangunkusumo National Central Hospital (RSCM), of whom 98 met the inclusion criteria. We excluded 28 patient medical records that had been permanently deleted, 12 medical records that could not be found, and 44 medical records that fulfilled the exclusion criteria. The flow of subjects throughout the study can be seen in Fig. 1.

The baseline characteristics of subjects are presented in Table 1. Bivariate regression analysis of subjects’ characteristics and CTO is shown in Table 2. All independent variables with *p*-value < 0.25 in the bivariate analysis were included in multivariate analysis.

**Table 1:** Characteristics of study subjects

Variables	(N = 98)
Gender, n (%)	
Male	89 (90.8)
Female	9 (9.2)
Median age, years (IQR)	53 (48–59)
Age, n (%)	
< 60 years	84 (85.7)
≥ 60 years	14 (14.3)
Median NLR (IQR)	6.83 (5.15–9.66)
Diabetes mellitus, n (%)	31 (31.6)
Median HbA1C, % (IQR)	5.9 (5.40–7.25)
Hypertension, n (%)	57 (58.2)
Mean SBP (SD)	125.94 (27.62)
Mean DBP (SD)	80.13 (17.46)
Dyslipidemia, n (%)	79 (80.6)
Median total cholesterol, mg/dL (IQR)	145 (116–190)
Mean LDL, mg/dL (SD)	182.97 (50,75)
Mean HDL, mg/dL (SD)	43.72 (9.95)
Mean triglycerides, mg/dL (SD)	141.63 (34.84)
Previous ACS, n (%)	10 (10.2)
Antiplatelet, n (%)	5 (5.1)
Statin, n (%)	10 (10.2)
Smoking, n (%)	83 (84.7)
Family history of CAD, n (%)	20 (20.4)
CTO, n (%)	76 (77.6)

**Table 2:** Bivariate analysis

Variable	CTO		PR (95%CI)	*p*
	Yes	No		
Age, n (%)				
≥ 60 years	12 (85.7)	2 (14.3)	1.125 (0.879–1.439)	0.730
< 60 years	64 (76.2)	20 (23.8)		
Gender, n (%)				
Male	72 (80.9)	17 (19.1)	1.820 (0.867–3.819)	0.025*
Female	4 (44.4)	5 (55.6)		
Diabetes mellitus, n (%)				
Yes	21 (67.7)	10 (32.3)	0.825 (0.631–1.080)	0.126*
No	55 (82.1)	12 (17.9)		
Hypertension, n (%)				
Yes	43 (75.4)	14 (24.6)	0.937 (0.758–1.159)	0.629
No	33 (80.5)	8 (19.5)		
Dyslipidemia, n (%)				
Yes	62 (78.5)	17 (21.5)	1.065(0.794–1.429)	0.760
No	14 (73.7)	5 (26.3)		
Previous ACS, n (%)				
Yes	7 (70.0)	3 (30.0)	0.893 (0.585–1.362)	0.689
No	69 (78.4)	19 (21.6)		
Antiplatelet, n (%)				
Yes	4 (80.0)	1 (20.0)	1.033 (0.656–1.6571)	0.893
No	72 (77.4)	21 (22.6)		
Statin use, n (%)				
No	66 (75.0)	22 (25.0)	0.750 (0.664–0.847)	0.111*
Yes	10 (100.0)	0 (0.0)		
Smoking, n (%)				
Yes	69 (83.1)	14 (16.9)	1.781 (1.025–3.095)	0.004*
No	7 (46.7)	8 (53.3)		
Family history of CAD, n (%)				
Yes	15 (75.0)	5 (25.0)	0.959 (0.725–1.269)	0.768
No	61 (78.2)	17 (21.8)		
Median NLR (IQR)	7.81 (6.38–11.85)	3.38 (2.32–5.43)	N/A	< 0.001^a^
NLR				
≥ 6.42	56 (91.8)	5 (8.2)	1.698 (1.359–2.311)	< 0.001*
< 6.42	20 (54.1)	17 (45.9)		

* Chi Square test;* Uji Fisher Exact

^a^Mann Whitney test

**Fig. 2:**
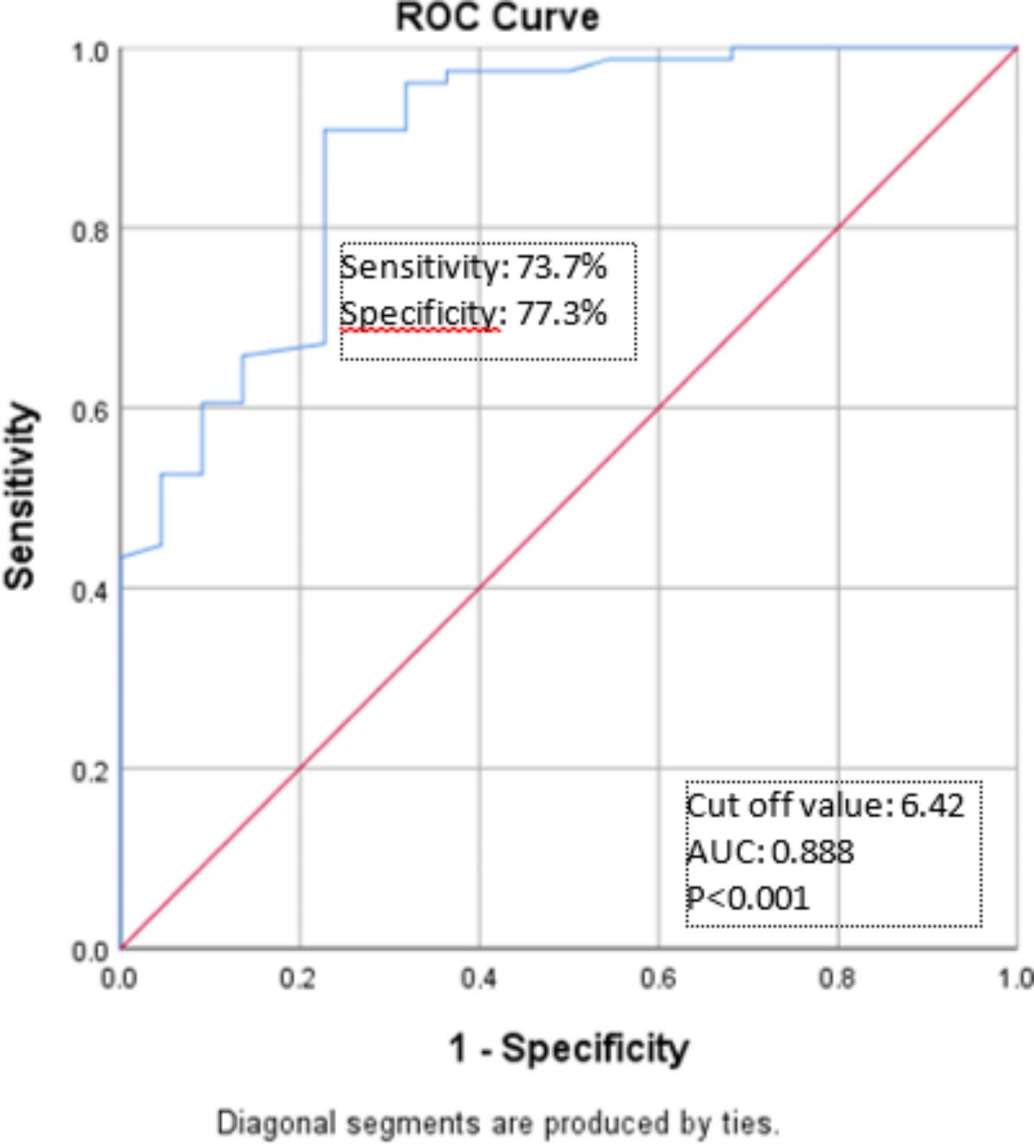
ROC curve analysis of NLR and CTO

An NLR cut-off point was determined by ROC curve analysis for NLR in patients with CTO. The AUC value of the ROC curve was 0.888 (95%CI 0.809-968), thus, the NLR cut-off point was 6.42, with 73.7% sensitivity and 77.3% specificity (Fig. 2). Chi-square analysis of NLR and CTO revealed that those with CTO had significantly higher NLR (≥ 6.42) compared to those not diagnosed with CTO (91.8% vs. 8.2%, respectively; *p* < 0.001). Higher NLR was found to increase the risk of CTO by 1.7-fold (95%CI 1.250–2.307). To evaluate the accuracy of NLR in predicting CTO, we calculated the positive predictive value (PPV). The PPV for NLR was 91% to predict CTO in patients with STEMI (Table 2).

Multivariate analysis of CTO and NLR, gender, DM, statin use, and smoking (*P* < 0.25 for all) was also performed. NLR retained a significant correlation with CTO; those with higher NLR value had 1.7 times higher risk of a CTO diagnosis (95%CI 1.234–2.221; *p* = 0.001). Smoking was also significantly correlated with CTO (95%CI 1.038–2.767; *p* = 0.035) (Table 3).

**Table 3:** Multivariate analysis

	Beta coefficient	S.E	Prevalence ratio (95% CI)	*p*-value
Smoking (yes)	0.528	0,734	1.695 (1.038–2.767)	0.035
NLR ≥ 6.42	0.504	0.432	1.656 (1.234–2.221)	0.001
Constant	-1.062	0.415	NA	0.038

## Discussion

This study, using register data of STEMI patients who underwent angiography procedures at RSCM in 2015–2018, revealed that 77.6% of such patients had CTO. This proportion was greater compared to that of other studies examining the prevalence of CTO in the 1990s, which ranged from 33 to 55%, depending on the definition of stenosis used [3, 4]. However, these older studies had several flaws, such as small sample size, retrospective design, and incomplete detailed information about the patients’ conditions. Newer studies, such as *The Canadian Multicenter Chronic Total Occlusion Registry* published in 2011, showed an even lower figure of 18.4%.^5^ However, that study included patients undergoing routine angiographic procedures, not just those in emergency conditions. In addition, their multicenter study was done for only a year [5]. Van Veelen et al., using data from the Dutch Heart Register reported in 2015–2018, stated that of all PCI procedures performed for non-emergency purposes, CTO was found in 6.3% of cases, with an increase from 5.9% in 2015 to 6.6% in 2018 [5]. A single center study in Canada involving all patients who underwent angiography found a CTO rate of 20% of patients diagnosed with CHD [11]. Differences in reported incidence from one study to another may have been due to different data collection methods. Our study used data from patients with STEMI conditions who then underwent an angiography. The baseline characteristics of subjects were similar to those in a study by Azzalini et al. [11]. On the other hand, studies that obtained lower CTO rates included patients who underwent routine angiography [5, 6].

The most important finding in our study was that in patients with CTO, the proportion who had NLR ≥ 6.42 (91.8%) was much higher than those with NLR < 6.42 (54.1%). This difference was statistically significant (*p* < 0.001) and supported by other research findings. Li et al. [12]. examined the association between NLR and the incidence of CTO and evaluated the clinical prognosis of patients undergoing primary percutaneous intervention. They measured NLR before and after the angiographic procedure, calculated the difference in NLR between the two measurements, and compared it to patients with and without CTO. At the time of admission, the NLR was higher in the CTO group, with a mean of 2.34 and a standard deviation of 1. In the group without CTO, the mean NLR was 1.81, with a standard deviation of 0.74. This mean difference was statistically significant (*p* < 0.001).^12^ This trend persisted in NLR values at admission, after angiography, and the difference in NLR before and after the procedure. NLR was significantly higher in the CTO group than in the group without CTO (*p* < 0.001).^12^ The difference in NLR was divided into two categories, namely, the difference below 0.5 and above 0.5 and compared to the clinical outcome at the time of hospital admission and when followed up at one year. They noted that the proportion of patients with an NLR difference above 0.5 had significantly more major cardiovascular events than those with a difference below 0.5 [12]. Furthermore, multivariate analysis revealed that NLR differences had an independent effect on the incidence of intrastent stenosis and major cardiovascular events in one year [12]. Zhang et al. evaluated the ability of NLR to predict the prognosis of patients presenting with STEMI during hospitalization and one year after the event in a meta-analysis. They assessed a variety of outcomes, but a significant outcome to be discussed in this section was the incidence of in-stent thrombosis. In-stent thrombosis in ACS patients following percutaneous coronary intervention procedures can completely occlude coronary arteries, the process of which is similar to that of coronary artery disease [13].

We found that NLR can predict the incidence of CTO in STEMI patients with 73.7% sensitivity, 77.3% specificity, and good discrimination ability (AUC 0.88; 95%CI 0.809–0.968). The cut-off value obtained was 6.42. These results are in agreement with other studies showing that NLR can be used as a predictor of CTO incidence. Demir et al. [7]. obtained a cut-off value of 2.09, with an AUC of 0.74 (95% CI 0.68–0.81), 61% sensitivity, and 69.3% specificity. Our NLR cut-off value had better discrimination ability and higher sensitivity. To the best of our knowledge, no other study has explicitly evaluated the ability of NLR biomarker to predict the incidence of CTO as expressed by the cut-off point, sensitivity and specificity, as well as the ability to discriminate against AUC values, ​​other than the study by Demir et al. [7]. However, a few studies evaluated the ability of the NLR to predict the severity or degree of CHD obstruction as assessed by Gensini or SYNTAX scoring, such as the meta-analysis conducted by Li et al. [14]. They reported a NLR cut-off point, AUC from an NLR ROC curve, and OR value from multivariate analysis. The meta regression showed that a useful NLR cut-off ranged from 1.95 to 3.97 and had a weak ability (AUC = 0.66; 95% CI 0.64–0.68) to predict the incidence of severe CHD, as defined by both Gensini and SYNTAX [12]. The meta-analysis also analyzed multivariate data from eleven studies and showed that a high NLR value was an independent risk factor (*p* < 0.001; OR 1.50; 95% CI 1.32–1.72 with heterogeneous data, I2 = 81%), which was able to predict the occurrence of severe blockage according to the definition of Gensini and SYNTAX [14].

In 2021, Yilmaz [15] also confirmed the findings of the meta-analysis by Li et al. [14]. Using SYNTAX scoring to categorize the severity of obstruction in patients diagnosed with CTO, they found that higher NLR scores were associated with higher scores. Also, higher SYNTAX correlated with more severe coronary artery blockage (*p* = 0.002).^15^ These findings confirmed that NLR was predictive of the occurrence of severe coronary artery occlusion. NLR has recently received special attention because it is potentially a biomarker to predict various cardiovascular events, including the incidence of CTO in patients with CHD [12]. Increased inflammatory markers and leukocyte count have been associated with the extent and severity of CHD, indicators of systemic inflammation, and higher mortality rates [16]. In addition, NLR has been correlated with the severity and complexity of CHD [17]. NLR at baseline is known to be independently associated with more complex CHD. Not only that, higher NLR has been correlated with the development of poor coronary collateral circulation in patients with CTO [18]. A meta-analysis included two studies that evaluated the incidence of post-interventional in-stent thrombosis in STEMI patients. Kaya et al. [19]. divided the NLR group into three categories: scores < 3.3, scores between 3.3 and 4.4, and scores > 4.4. On the other hand, Akpek et al. [20]. divided the NLR into two categories, with a cut-off of 3.3. The results of these two studies differed from our results, as Kaya et al. and Akpek et al. noted that higher NLR could significantly predict the incidence of in-stent thrombosis (*p* < 0.001 for both) [18, 19]. A pooled analysis of these two studies revealed that a higher NLR could significantly predict the incidence of in-stent thrombosis (RR 2.72; 95%CI 1.66–4.44; *p* < 0.001) with homogeneous data (I2 = 0%).^13^ Ischemia can trigger several inflammatory processes and ultimately change the composition of leukocytes. Neutrophils play a major role in endothelial damage and platelet aggregation during ACS initiation events by releasing pro-oxidant and prothrombotic substances [12]. Meanwhile, lymphocytes have been shown to be an important factor in atherosclerosis by controlling and regulating the inflammatory response [12]. Because this inflammation is continuous, NLR is thought to play an important role in predicting the outcome in the process of CHD pathogenesis. Several early studies examining the role of NLR found that this biomarker could predict several cardiovascular diseases, especially CHD. Significant associations were noted between NLR and severity of coronary artery disease, as assessed by the Gensini and Syntax scores [16, 20]. These two scoring systems are based on coronary angiography results (gold standard).

Few studies have evaluated the role of NLR on the incidence of chronic total occlusion because its pathogenesis is more complex than other acute cardiovascular events. CTO is formed due to continuous cellular inflammation and neovascularization of blood vessel walls so that thrombus formation, inflammatory cell recruitment, smooth muscle cell migration, and extracellular matrix deposition occur continuously. As such, NLR was found to be significantly higher in patients with CTO. Therefore, considering these benefits, NLR can be a quick and inexpensive screening tool to predict the incidence of CTO in patients with previous CHD.

In addition to NLR, we also found that smoking was significantly associated with the incidence of CTO. In CTO patients, the proportion who smoked (83.1%) was much greater than that of non-smokers (46.7%) (*p* = 0.004). Similarly, there were consistently more smokers in the CTO group than in the non-CTO group in studies by Demir et al. [7]. and Fefer et al. [5]. (40% vs. 28% and 24% vs. 16%, respectively; *p* < 0.001 for both). Smoking is also a known risk factor since the Framingham Heart Study [16]. Smokers are at an increased risk of myocardial infarction or sudden death and that risk is related to the number of cigarettes smoked each day [21].

### Study limitation

This study used a cross-sectional design, in which observations were done in only one period of time, thus temporal relationships between the various risk factors and the outcomes assessed were not taken into account. In addition, very specific patient characteristics may not be representative of all CTO patients who do not manifest as STEMI. Moreover, this research was conducted only at one center, so care should be taken in extrapolating to a wider context.

## Conclusion

In conclusion, the proportion of CTO among patients with STEMI who underwent coronary angiography in RSCM between the period of 2015–2018 was 77.6%. Multivariate analysis revealed that smoking and NLR had associations with the occurrence of CTO in STEMI cases. In the future, an NLR cut-off value of ≥ 6.42 can potentially be used as a marker for CTO occurrence, with 73.7% sensitivity, 77.3% specificity, and 91% PPV. The use of NLR as CTO marker in the future should be assessed further by a multicenter longitudinal study to apply this result to a larger population.

### Electronic supplementary material

Below is the link to the electronic supplementary material.

**Supplementary Material 1: Supplementary Table 1.** Dataset of The Study

## Data Availability

All data generated or analysed during this study are included in this published article [and its supplementary information files].
